# Central Venous Catheter-Related Infection in Severe Trauma Patients

**DOI:** 10.1007/s00268-015-3137-y

**Published:** 2015-07-03

**Authors:** Seok Hwa Youn, John Cook-Jong Lee, Younghwan Kim, Jonghwan Moon, Younghwa Choi, Kyoungwon Jung

**Affiliations:** Division of Trauma Surgery, Department of Surgery, Ajou University School of Medicine, 206 World cup-ro, Yeoungtong-gu, Suwon, 443-749 Korea; Division of Infectious Disease, Department of Internal Medicine, Ajou University School of Medicine, Suwon, Korea

## Abstract

**Aim:**

To evaluate the CVC-related infection rate according to catheter insertion site and to analyze the risk factors for catheter-related local infections (CRLI) and bloodstream infections (CRBSI) among severe trauma patients.

**Methods:**

We reviewed the medical records of 736 severe trauma patients with an Injury Severity Score of >15. Poisson regression was used to compare the infection rates according to the catheter insertion sites. Univariate analysis of the groups with and without CVC-related infection was used to identify confounding variables for inclusion in multivariate models that were used to identify the risk factors for CRLI and CRBSI.

**Results:**

We evaluated 1646 catheter insertions and their duration of insertion and found 1241 subclavian (18,461 days), 251 internal jugular (3454 days), and 154 femoral catheters (1526 days). The CRLI infection rate per 1000 catheter days was significantly lower for subclavian, compared to that for internal jugular (4.83 vs. 9.55, respectively; *P* < 0.001) and femoral catheters (4.83 vs. 7.93, respectively; *P* = 0.013). Multivariate logistic regression analysis revealed that catheter insertion duration [odds ratio (95 % confidence interval): 1.035 (1.021–1.050), *P* < 0.001] and subclavian access [0.532 (0.366–0.775), *P* < 0.001] were significantly associated with CRLI, while catheter insertion duration [1.024 (1.002–1.046), *P* = 0.032] was significantly associated with CRBSI.

**Conclusions:**

To reduce the rate of CVC-related infections in severe trauma patients, we suggest that catheters be shifted from the internal jugular or femoral veins to the subclavian vein as soon as possible and that the duration of catheter insertion should be minimized.

## Introduction

Central venous catheters (CVCs) are inserted for severe trauma patients who are hemodynamically unstable at the time of their admission to the trauma department. Other indications for CVCs include resuscitations and patients with a deteriorating condition after surgical or radiological interventions. In this context, CVCs provide many benefits in the acute and critical care fields, including hemodynamic monitoring, fluid resuscitation, massive transfusion, administration of medication, and nutritional support [1, 2]. However, despite these benefits, CVCs are associated with various complications, including infections, hemorrhage, pneumothorax, arterial puncture, and thrombosis. Among these complications, CVC-related infections are the most studied, as they can significantly influence the length of intensive care unit (ICU) stays, hospital costs, and mortality rates (up to 25 %) [3–6]. Various studies of CVC-related infections in the critical care setting have been reported, and various treatment guidelines have been developed based on their findings, including the Centers for Disease Control’s (CDC) guidelines. However, while many studies have evaluated CVC-related infections in critically ill patients, few studies have evaluated these infections in severe trauma patients. Therefore, this study aimed to evaluate infection rate according to catheter insertion site and to analyze the risk factors for CVC-related infection among severe trauma patients.

## Materials and methods

This observational study was conducted at Ajou University Hospital (Suwon, Korea), which is a leading tertiary hospital in Korea, with an annual emergency department volume of 16,000 trauma patients, many of whom have an ISS of >15. Most polytrauma patients, except those with isolated brain or solitary orthopedic injuries, are admitted to the Division of Trauma Surgery. Between January 2009 and December 2013, 1178 patients were admitted to our department via the emergency room, including 736 patients who had an ISS of >15. Among these 736 patients, 698 patients received CVCs, including 56 patients who subsequently died. However, we did not censor their data, because we inspected the insertion site at every dressing time, even on the day of death. In contrast, we excluded all cases involving peripherally inserted central catheters, as their insertions were away from the three sites of interest. This study’s design was reviewed and approved by the institutional review board of Ajou University Hospital (IRB No. MED-OBS-14-402).

The most common indications for CVC insertion were hemodynamic instability due to trauma and total parenteral nutrition after admission. The primary access site was the subclavian vein, with the internal jugular and femoral veins serving as secondary access sites, although the physician who inserted the catheter made the final decision at the time of the insertion. However, given the general features of trauma patients, it was not always possible to use the primary access site, based on nearby injuries.

Maximum full barrier protection was used whenever possible, although these protocols occasionally could not be followed in cases that required urgent care. The CVCs that were inserted during urgent care were removed within 72 h (as soon as possible). All catheters were coated in antibiotics, and ultrasonography was occasionally used to guide the insertion, based on the physicians’ discretion. After insertion, a disposable sterile gauze dressing or transparent dressing was applied, although no antibiotic cream or lotion was applied around the insertion site. While the catheter was maintained, the line connections were strictly controlled to avoid contamination, and strict hand hygiene was observed before and after catheter manipulation. We removed the catheter when the indication for the insertion had resolved, or when signs of local infection were observed when changing the dressing. In the present study, fever with an unknown cause or an insertion duration of >2 weeks were considered indications for removal, even in the absence of other infection signs. In addition, based on previous studies, femoral catheters were immediately moved to the subclavian (first priority) or internal jugular (second priority) veins when those sites became accessible, even if there were no signs of infection.

We reviewed the patients’ general information from their medical charts, including sex, age, ISS, length of ICU stay, duration of catheter insertion, catheter insertion sites, catheter types, reasons for catheter removal, CVC-related infection, and related microorganism. Catheter types were classified as single-lumen, double-lumen, and triple-lumen catheters according to the number of lumens. Single-lumen catheters included the ARROW^®^ CVC (Arrow, Reading, PA, USA) and the IntroFlex™ (Edwards Lifesciences, Irvine, CA, USA); double-lumen catheters included the ARROW^®^ CVC, the ARROW^®^ MAC, and the MAHURKAR™ (11.5-Fr dual-lumen catheters for acute dialysis; Covidien, Mansfield, MA, USA); and the triple-lumen catheters were the ARROW^®^ CVCs. CVC-related infections were divided into catheter-related local infections (CRLI) and bloodstream infections (CRBSI). We defined CRLI as local signs of infection at the insertion site, including erythema, local pain, inflammation, or purulent discharge, with microorganism colonization of the catheter tip. CRBSI was defined as the presence of signs of systemic infection with positive culture results from the peripheral venous blood and catheter tip. We diagnosed CRBSI based on the presence of >15 colony-forming units in the catheter tip culture and after excluding other non-catheter sources of infection.

SPSS software (version 21.0, IBM Corp, Armonk, NY) was used for all statistical analyses. Poisson regression distribution with general log-linear analysis was used to compare the infection rates and determine the risk of infection according to the catheter insertion sites. Variables were reported as median (range) or number (percentage), and the *χ*^2^, Fisher’s exact, or Mann–Whitney *U* test was used, as appropriate. To identify the variables confounding the risk factors for catheter-related infection, univariate analysis was used to compare the groups with and without infection. Multivariate modeling was performed using conditional logistic regression with a backward stepwise elimination procedure, and variables were selected for inclusion in the model based on the results of the univariate analysis (*P* value <0.1). For all other tests, a *P* value of <0.05 was used to determine statistical significance.

## Results

Among the 736 (62.5 %) severe trauma patients with an ISS of >15, 556 patients (75.5 %) were men and the median age was 48 (3–94) years. The median ISS was 22 (16–75) and the median length of the ICU stay was 11 (0–190) days. A total of 1646 CVCs were inserted into the subclavian (*n* = 1241), internal jugular (*n* = 251), and femoral (*n* = 154) veins. The total duration for subclavian catheters was 18,461 days (median, 13 days; range 1–99 days), compared to 3454 days (12; 1–63) for internal jugular catheters and 1526 days (5; 1–52) for femoral catheters (Table 1).

**Table 1:** Catheter-related infections according to the insertion site

Insertion site	Number of catheters	Catheter duration (days)		Number of CRLIs		Number of CRBSIs	
		Total	Median (IQR)	Total (% CVC)	Per 1000 catheter days	Total (% CVC)	Per 1000 catheter days
Subclavian	1241	18,461	13 (7–21)	91 (7.33)	4.83	43 (3.46)	2.28
Internal jugular	251	3454	12 (6–20)	33 (13.15)	9.55	12 (4.78)	3.47
Femoral	154	1526	5 (3–15)	15 (9.74)	7.93	3 (1.96)	0.61
Total	1646	23,441	12 (6–20)	139 (8.44)	5.75	58 (3.52)	2.44

*CRLI* catheter-related local infection, *CRBSI* catheter-related bloodstream infection, *IQR* interquartile range, *CVC* central venous catheter

We observed 139 cases of CRLI and 58 cases of CRBSI, with rates of 5.75 and 2.44 per 1000 days, respectively. The subclavian vein was involved in 91 CRLI episodes (4.83 per 1000 days), compared to 33 episodes (9.55 per 1000 days) in the internal jugular vein and 15 episodes (7.93 per 1000 days) in the femoral vein. The subclavian vein had a significantly lower CRLI rate, compared to the internal jugular and femoral veins [odds ratio (OR) 0.516, 95 % confidence interval (CI) 0.346–0.768, *P* = 0.001, and OR 0.501, 95 % CI 0.290–0.866, *P* = 0.013, respectively]. However, the internal jugular and femoral veins did not exhibit a significant difference in their CRLI rate (OR 0.972, 95 % CI 0.528–1.789, *P* = 0.927) (Table 2). The subclavian vein was involved in 43 CRBSI episodes (2.28 per 1000 days), compared to 12 episodes (3.47 per 1000 days) in the internal jugular and three episodes (0.61 per 1000 days) in the femoral veins. However, these differences were not significantly different when we compared the subclavian and internal jugular (OR 0.670, 95 % CI 0.354–1.271, *P* = 0.221), the subclavian and femoral (OR 1.185, 95 % CI 0.368–3.819, *P* = 0.776), or the internal jugular and femoral veins (OR 1.767, 95 % CI 0.499–6.262, *P* = 0.378) (Table 3).

**Table 2:** Comparison of central venous catheter-related local infections according to the insertion site

Insertion site	Number of infections per 1000 catheter days	OR (95 % CI)	*P* value
Subclavian versus internal jugular	4.83 vs. 9.55	0.516 (0.346–0.768)	0.001
Subclavian versus femoral	4.83 vs. 7.93	0.501 (0.290–0.866)	0.013
Internal jugular versus femoral	9.55 vs. 7.93	0.972 (0.528–1.789)	0.927

*OR* odds ratio, *CI* confidence interval

**Table 3:** Comparison of central venous catheter-related bloodstream infections according to the insertion site

Insertion site	Number of infections per 1000 catheter days	OR (95 % CI)	*P* value
Subclavian versus internal jugular	2.28 vs. 3.47	0.670 (0.354–1.271)	0.221
Subclavian versus femoral	2.28 vs. 0.61	1.185 (0.368–3.819)	0.776
Internal jugular versus femoral	3.47 vs. 0.61	1.767 (0.499–6.262)	0.378

*OR* odds ratio, *CI* confidence interval

When we evaluated the CRBSI cases, we observed 58 causative microorganisms, including 32 gram-positive bacteria, 10 gram-negative bacteria, and 17 yeast strains. Coagulase-negative *Staphylococci* were involved in 21 cases, *Staphylococcus aureus* was involved in 9 cases, *Acinetobacter baumannii* in 5 cases, *Enterococcus faecalis* in 2 cases, *Serratia marcescens* in 2 cases, and *Klebsiella pneumonia* in 2 cases; no cases were involved *Pseudomonas* infection. Among the yeast infections, *Candida parapsilosis* was involved in 9 cases, *Candida albicans* in 6 cases, and *Candida pelliculosa* and *Candida intermedia *in 1 case each (Table 4).

**Table 4:** Microorganisms responsible for central venous catheter-related bloodstream infections

Microorganism	Number	Percent (%)
Gram positive	32	55.2
Coagulase-negative *Staphylococci*	21	36.2
*Staphylococcus aureus*	9	15.5
*Enterococcus faecalis*	2	3.4
Gram negative	9	15.5
*Acinetobacter baumannii*	5	8.6
*Serratia marcescens*	2	3.4
*Klebsiella pneumonia*	2	3.4
Yeast	17	29.3
*Candida parapsilosis*	9	15.5
*Candida albicans*	6	10.3
*Candida pelliculosa*	1	1.7
*Candida intermedia*	1	1.7
Total	58	100.0

In the univariate analysis, length of ICU stay, catheter insertion duration, catheter insertion site (subclavian), and total parenteral nutrition (TPN) administration were associated with CRLI (*P* < 0.1) (Table 5). Although TPN administration was found to be a significant factor, it was not included in multivariate analysis because the MAC and MAHUKAR catheters (which comprised a large portion of the CVCs) cannot accommodate TPN administration. Multivariate analysis revealed that catheter insertion duration (OR 1.035, 95 % CI 1.021–1.050, *P* < 0.001) and subclavian access (OR 0.532, 95 % CI 0.366–0.775, *P* < 0.001) were significantly associated with CRLI (Table 6). Similar analysis for CRBSI identified length of ICU stay, catheter insertion duration, and number of catheter lumens as potential associated factors, although only catheter insertion duration (OR 1.024, 95 % CI 1.002–1.046, *P* = 0.032) was significantly associated with CRBSI in the multivariate analysis (Table 7).

**Table 5:** Univariate analysis of the confounding variables associated with central catheter-related infection

Variable	CRLI: yes (*n* = 139)	CRLI: no (*n* = 1507)	*P* value	CRBSI: yes (*n* = 58)	CRBSI: no (*n* = 1588)	*P* value
Age (years)	52 (3–94)	51 (8–86)	0.657	51 (16–84)	51 (3–94)	0.541
Sex, % male	79.9 % (111)	77.2 % (1163)	0.469	81.0 % (47)	77.3 % (1227)	0.500
ICU length of stay (days)	35 (0–148)	21 (0–190)	<0.001	32 (4–148)	22 (0–190)	0.033
Catheter insertion duration (days)	16 (1–53)	11 (1–99)	<0.001	17.5 (5–37)	12 (1–99)	<0.001
Injury Severity Score	25 (16–50)	25 (16–75)	0.953	25 (16–50)	25 (16–75)	0.730
Catheter insertion sites			0.009			0.316
Subclavian	65.5 % (91)	76.3 % (1,150)		74.1 % (43)	75.4 % (1198)	
Internal jugular	23.7 % (33)	14.5 % (218)		20.7 % (12)	15.1 % (239)	
Femoral	10.8 % (15)	9.2 % (139)		5.2 % (3)	9.5 % (151)	
Catheter type^a^			0.266			0.036
Single-lumen	4.3 % (6)	5.8 % (87)		1.7 % (1)	5.8 % (92)	
Double-lumen	34.5 % (48)	40.1 % (605)		27.6 % (16)	40.1 % (637)	
Triple-lumen	61.2 % (85)	54.1 % (815)		70.7 % (41)	54.1 % (859)	
Total parenteral nutrition			<0.001			<0.001
Yes	82.0 % (114)	60.6 % (904)		91.4 % (53)	60.8 % (965)	
No	18.0 % (25)	40.0 % (603)		8.6 % (5)	39.2 % (623)	
Transfusion			0.830			0.657
Yes	74.1 % (103)	73.3 % (1,104)		75.9 % (44)	73.2 % (1,163)	
No	25.9 % (36)	26.7 % (403)		24.1 % (14)	26.8 % (425)	

Data are presented as median (range) or  % (*n*)

*CRLI* catheter-related local infection, *CRBSI* catheter-related bloodstream infection, *ICU* intensive care unit

^a^Catheter types are classified according to the number of catheter lumens

**Table 6:** Multivariate analysis of the confounding variables that were associated with central catheter-related local infection

Model	Covariates	OR (95 % CI)	*P* value
1	ICU length of stay (days)	1.004 (0.999–1.008)	0.135
	Catheter insertion duration (days)	1.032 (1.017–1.047)	<0.001
	Catheter insertion site—subclavian	0.549 (0.376–0.801)	0.002
2	Catheter insertion duration (days)	1.035 (1.021–1.050)	<0.001
	Catheter insertion site—subclavian	0.532 (0.366–0.775)	0.001

*OR* odds ratio, *CI* confidence interval, *ICU* intensive care unit

**Table 7:** Multivariate analysis of the confounding variables that were associated with central catheter-related bloodstream infection

Model	Covariates	OR (95 % CI)	*P* value
1	ICU length of stay (days)	1.003 (0.996–1.010)	0.423
	Catheter insertion duration (days)	1.021 (0.998–1.045)	0.075
	Number of catheter lumens	1.738 (1.019–2.963)	0.042
2	Catheter insertion duration (days)	1.024 (1.002–1.046)	0.032
	Number of catheter lumens	1.696 (0.998–2.883)	0.051

*OR* odds ratio, *CI* confidence interval, *ICU* intensive care unit

## Discussion

Previous studies that have analyzed CRLI in multidisciplinary ICUs have reported incidences of 6–15 % and infection rates of 1.47–6.05 per 1000 days [1, 7]. In the present study, we observed similar, although slightly higher, incidences and infection rates (7.07 % and 5.75 per 1000 days, respectively). However, only a few previous studies have evaluated CRLI, using various clinical diagnostic criteria, and more organized prospective studies are needed to provide a more accurate estimation of the incidence and infection rate.

In contrast, numerous studies have evaluated CRBSI, with reported incidences varying from 1 to 13 % [1, 7–19]. According to the National Nosocomial Infections Surveillance (NNIS) report in 2004, the CRBSI rate from various ICUs was 1.8–5.2 per 1000 days [20], and the highest rate was observed in a trauma ICU. Happily, another study has reported that the CRBSI rate decreased each year between 2005 and 2010 [21], and additional studies have reported decreases to 2.05 per 1000 days (in a 2009 study) and 0.4 per 1000 days (in a study of 21,259 catheter days) [22, 23]. These decreasing CRBSI rates are thought to be related to the strict use of full barrier protection, antibiotic-coated catheters, ultrasonography, and thorough catheter management [24, 25]. Although the present study only evaluated severe trauma patients, while the previous studies have evaluated multidisciplinary ICUs and trauma ICUs using the NNIS guidelines, we observed a slightly lower CRBSI rate of 2.4 per 1000 days. This difference may be related to our strict institutional adherence to the NNIS guidelines, which we believe has reduced our CRBSI rate.

There is currently debate regarding the optimal CVC insertion site for lowering the CRBSI rate, and various guidelines have been released, based on previous studies. However, few studies have evaluated this topic for CRLI, although a study by Lorente et al. revealed that the highest CRLI rate occurred in the femoral vein, while the lowest rate occurred in the subclavian vein [1]. Regarding the CRBSI rate, several studies have found that the femoral access has the highest rate [1, 7, 13, 26, 27], while other studies have found that the internal jugular access has the highest rate [17, 18, 28]. However, the internal jugular access and subclavian access have been compared in other studies, and those studies have reported that the jugular access has a higher CRBSI rate compared to the subclavian access [9, 14, 29]. Furthermore, the CDC guidelines have recommended the optimal access site order (subclavian, internal jugular, and femoral vein, respectively) in “recommendation 1A,” which is based on data from several large studies. However, Marik et al. have stated that recommendation 1A may not be supported by the results of various studies; therefore, even the current guidelines cannot be considered a consensus opinion [21, 24, 30]. Moreover, those studies evaluated patients who were in critical condition at multidisciplinary ICUs (not necessarily for trauma-related reasons), and their results may not be relevant to the trauma setting, where the patient’s condition may limit access site availability and increase the risk of infection via contaminated wounds.

Our results indicate that only the use of the subclavian vein significantly affected the CRLI rate, although no significant differences were observed in the CRBSI rates when we compared the three sites. However, the inguinal area has a high density of local skin flora, which can likely increase that site’s infection rate, although we have markedly reduced the chances of infection at the femoral access by implementing the procedural suggestions from previous studies. When femoral access was unavoidable, we transferred the catheter to the other sites as soon as possible to decrease the catheter insertion duration and infection rate. Based on that process, the femoral vein appears to have a superior CRBSI rate, although this difference was not statistically significant. This finding may be related to the fact that we removed the femoral catheter as soon as possible, as catheter insertion duration was positively associated with both the CRLI and CRBSI rates. Furthermore, the higher infection rate at the internal jugular access (vs. the subclavian access) may be related to an increased likelihood of contamination from the oral cavity and tracheostomy sites (due to proximity), or to the presence of skin creases around the jugular access, which made it difficult to maintain an appropriate position for the gauze dressing.

Regarding the microorganisms that were responsible for CRBSI, our findings are similar to those of previous studies, which have reported that gram-positive bacteria are the most common, although we, and other investigators, have observed that yeasts are more common than gram-negative bacteria [20, 31]. In our center, third-generation cephalosporin is typically used for the initial antibiotic treatment for severe trauma patient, and the wide antibiotic spectrum may be partially responsible for our findings. Among hospital-acquired infections, the rate of fungemia has increased over the past two decades, and several studies have reported that this increase is related to the increased use of broad-spectrum antibiotics [32]. Furthermore, once yeast appears in the blood, which can adhere to the catheter tip under the fibrous protein membrane, thereby escaping the patient’s immune system and any anti-fungal agents, and subsequently contribute to the CRBSI rate [33]. This mechanism may explain the higher rate of yeast or multidrug-resistant microorganisms that were related to CRBSI.

There are several limitations in our study. First, our study used a retrospective design. Second, the insertion sites were not randomly assigned and were selected based on existing treatment guidelines. Third, we did not use the CDC’s definition of CRBSI (“differential period of CVC culture versus peripheral blood culture positivity of more than 2 h”), as our blood and catheter tip culture system was not designed for this comparison. Therefore, it is possible that our findings are different from those of previous studies that followed the CDC guidelines. Fourth, we only assessed infectious complications, as complete data regarding iatrogenic complications (e.g., pneumothorax, arterial puncture, or thrombosis) were not available. Nevertheless, the occurrence of these complications might influence the selection of an appropriate catheter insertion site.

## Conclusions

Although selection of the CVC site was limited in severe trauma patients, our results suggest that catheter insertion through the subclavian vein may reduce the CVC-related infection rate in severe trauma patients, compared to insertion through the internal jugular or femoral veins. In addition, catheter insertion duration is significantly associated with the rate of central catheter-related infection. Therefore, this study confirms that the subclavian vein should be selected first, followed by the internal jugular vein, and the femoral vein as the last option. Although severe trauma may necessitate the use of the femoral vein, we suggest moving catheters from the femoral vein to the subclavian vein as quickly as possible; the internal jugular vein might also be needed in these cases. Furthermore, we recommend that the CVC be completely removed as soon as it is no longer indicated. However, further prospective studies are needed to confirm these findings and recommendations.
